# Comparative Transcriptomic Analysis of the Interaction between *Penicillium expansum* and Apple Fruit (*Malus pumila* Mill.) during Early Stages of Infection

**DOI:** 10.3390/microorganisms7110495

**Published:** 2019-10-28

**Authors:** Kaili Wang, Xiangfeng Zheng, Xiaoyun Zhang, Lina Zhao, Qiya Yang, Nana Adwoa Serwah Boateng, Joseph Ahima, Jia Liu, Hongyin Zhang

**Affiliations:** 1 School of Food and Biological Engineering, Jiangsu University, Zhenjiang 212013, China; 15981843773@163.com (K.W.); zhangxiaoyungu@126.com (X.Z.); linazhao@ujs.edu.cn (L.Z.); yangqiya1118@163.com (Q.Y.); boatengn70@yahoo.com (N.A.S.B.); Joseph_ahima@yahoo.com (J.A.); 2 School of Food Science and Engineering, Yangzhou University, Yangzhou 225009, China; 18796002921@163.com; 3 Chongqing Key Laboratory of Economic Plant Biotechnology, College of Landscape Architecture and Life Science/Institute of Special Plants, Chongqing University of Arts and Sciences, Yongchuan, Chongqing 402160, China

**Keywords:** cell wall degradation enzymes, ETI, *Penicillium expansum*-Apple interaction, pH regulation, plant hormone signaling, PTI

## Abstract

Blue mold, caused by *Penicillium expansum*, is an important postharvest disease of apple, and can result in significant economic losses. The present study investigated the interaction between *P. expansum* and wounded apple fruit tissues during the early stages of the infection. Spores of *P. expansum* became activated one hour post-inoculation (hpi), exhibited swelling at 3 hpi, and the germ tubes were found entering into apple tissues at 6 hpi. RNA-seq was performed on samples of *P. expansum* and apple fruit tissue collected at 1, 3, and 6 hpi. The main differentially expressed genes (DEGs) that were identified in *P. expansum* were related to interaction, cell wall degradation enzymes, anti-oxidative stress, pH regulation, and effectors. Apple tissues responded to the presence of *P. expansum* by activating pathogen-associated molecular pattern (PAMP)-triggered immunity (PTI) at 1 hpi, then activated effector-triggered immunity (ETI) at 3 hpi. This research provides new information on the interaction between *P. expansum* and apple fruit tissue at an early stage of the infection process.

## 1. Introduction

Apple is one of the major fruit crops produced worldwide and can be stored for long periods of time under proper conditions, to ensure a sufficient supply of fresh fruits all year round [1]. Apple fruit possesses a high level of nutrients, making them susceptible to postharvest diseases during processing and storage [2]. *Penicillium expansum*, commonly referred to as blue mold, is the most common postharvest pathogen on apples and can cause up to a 50% loss in marketable fruit [3]. *P. expansum* also produces patulin, which is an immunotoxin, hepatotoxin, and neurotoxin in humans and animals [3].

The use of chemical fungicides remains the principle method of managing postharvest diseases in apple and other fruits [4]. However, regulatory and public concerns have increasingly been raised about the effect of synthetic chemicals on human health and the environment [5]. Although biological control has been reported to be an alternative to the use of synthetic chemical fungicides [6], they are not as effective. Therefore, the need to develop effective biologically-based alternatives has fostered studies directed at better understanding the molecular interactions that occur between postharvest pathogens and their hosts.

*P. expansum* is a necrotrophic pathogen that infects tissues through wounds or other entry ports in fruit tissues [7]. The transcriptomic analysis of the interaction between *P. expansum* and a susceptible resistant apple genotypes at 24, 48 and 72 h post-inoculation (hpi) has previously conducted [8]. Necrotrophic pathogens, however, may initiate their infection process immediately after spores come into contact with wounded tissues [9]. Therefore, earlier time points in the interaction between *P. expansum* and apple tissues still need to be examined.

Several studies have been conducted on the interaction between *P. expansum* on apple, and these include the response of apple to *P. expansum* and a non-pathogen, the effects of fruit maturity on susceptibility, the role of reactive oxygen species (ROS) and the identification of putative secreted effectors produced by *P. expansum.* However, no transcriptomic studies have been conducted in the early stages (0–6 hpi) of the infection process. Therefore, in the present study, high-throughput sequencing of the apple and fungal transcriptome were conducted during the initial stages of the infection process. RT-qPCR was used to confirm the accuracy of the transcriptome data. This approach provided details about the transcriptional changes that occur in *P. expansum* and apple during the first six hours of the infection process. Differentially expressed genes (DEGs) and their associated metabolic pathways were identified for different biological processes in both the pathogen and fruit.

## 2. Materials and Methods

### 2.1. Fruit

Apple (*Malus pumila* Mill, Yantai, China) fruits used in this study were selected at commercial maturity with uniformity in appearance and size, and the absence of any infections or injuries. The fruits were disinfected by immersion in 0.2% (*w*/*v*) NaClO for 2 min, washed in water, and then air dried.

### 2.2. Pathogen

*P. expansum* strain was originally isolated from rotten apple fruits. Following the methodology of Yan et al. for culturing the *P. expansum* fungi [10]. Afterwards, spore suspensions were prepared and adjusted to the required concentration with sterile distilled water. The spores at this stage were re-suspended with a vortex and considered to be resting spores [11].

### 2.3. Inoculation of Apples with P. expansum

Three wounds (5-mm-deep and 4-mm-diameter) were made with a sterile corkborer around the equator of each fruit. Then, 30 μL of the spore suspension (4 × 10^8^ spores/mL) of *P. expansum* were inoculated into each apple wound. The treated apples were then put in enclosed plastic baskets to maintain a 95% humidity and incubated at 25 °C.

### 2.4. Microscopy

Apples wounds were inoculated with resting spore suspensions of *P. expansum*, and the establishment of the spores was monitored with a Pannoramic MIDI slide scanner (3DHISTECH Kft., Budapest, Hungary) to determine the key time points to collect samples for the transcriptomic analysis. Sections of the inoculated apple wounds were made with a scalpel and stained with carbonic acid lactate cotton blue stain for 2 min. Excess dye fluid was then absorbed with a piece of filter paper. A drop of neutral gum sealant was then placed on the sample, followed by a coverslip, which was then pressed gently until the excess neutral gum was discharged from under the coverslip. The slide was then cleaned with xylene to remove excess neutral gum on the edge of the coverslip. The infection process was observed at different time points of (0, 1, 3, 6, 9, 12 hpi) with the aid of a Pannoramic MIDI slide scanner. The infection process was examined twice using three biological replicates (individual apples) during each observation.

### 2.5. RNA Extraction

Tissue samples from wounded, inoculated apples were collected 1 hpi, 3 hpi, and 6 hpi. Samples taken at 0 hpi were used as a control. RNA was extracted strictly according to the operation instructions of Spin Column Fungal Total RNA Purification Kit (Sangon. Co., Shanghai, China). The inoculated apple wound tissues (approximately 2 g) were immediately immersed in liquid nitrogen and ground into a fine powder. Ground tissue samples were placed at −80 °C until further processing. Adsorption columns and collection pipes were used to purify the RNA. The quality of RNA was measured using a One Drop OD-1000+ spectrophotometer (Wuyi Science and Technology Co., Ltd., Nanjing, China). RNA was extracted from the wounds of three independent biological replicates for each time point. Each group was composed of 3 apples.

### 2.6. RNA-Seq Library Construction and Illumina Sequencing

Each RNA sample of apple wound tissues treated at different hours post-inoculation (0, 1, 3, 6 hpi) were used to conduct RNA-seq with three microgram high-quality RNA of each sample used for cDNA library. Enrichment of mRNA was performed through magnetic Oligo (dT) beads. RNA sequencing libraries were produced with the NEBNext^®^ Ultra RNA Library Prep kit for Illumina (New England Biolabs, Ipswich, MA, USA) with multiplexing primers, strictly according to the manufacturer’s instructions. The Agilent 2100 bioanalyzer and ABI StepOnePlus Real-Time PCR System were used to quantify and qualify the cDNA sample library, respectively. Finally, the library was sequenced via the Illumina HiSeq X Ten with the pair-end mode (performed by Genepioneer Biotechnologies Company) [12]. RNA-Seq was conducted only once.

### 2.7. Bioinformatic Analysis of the RNA-Seq Data

Raw reads were pre-processed to obtain the clean reads. The high quality clean sequence reads of *P. expansum,* and apple tissues were used in the downstream bioinformatic analysis. The reference genome of *P. expansum* (taxid: 27334) and apple (taxid: 3749) were used to align the paired-end clean reads. |log2 (Fold change)| ≥1 and false discovery rate (FDR) < 0.05 were considered as significantly differential expression. DEGs were assigned by comparing the gene expression level of 1 hpi, 3 hpi and 6 hpi to the control (0 hpi). The Gene Ontology and Kyoto Encyclopedia of Genes and Genomes classification were used for GO analysis and KEGG metabolic pathway analysis of DEGs. *P*-values < 0.05 were defined as significantly enriched.

The clustered profiles of DEGs with *p*-values < 0.05 were considered as significantly different from the reference set (the DEGs of 0 hpi). Two (2) profiles (profile 1, profile 9) in *P. expansum* and 3 profiles (profile 9, profile 1, profile 4) in apple were determined significantly different from the reference set.

### 2.8. Validation of RNA-Seq Data by RT-qPCR

RT-qPCR analysis was conducted using samples collected from wounded inoculated apples at 1, 3, and 6 hpi. Primers were designed and synthesized by Sangon Biotech. The gene specificity primers used in this study were listed in Table S1. RT-qPCR analysis was performed on an ABI PRISM 7500 Real-Time PCR System (Applied Biosystems, Foster, CA, USA). A *β-tublin* gene from *P. expansum* and an *actin* gene from apple were used as internal controls to normalize the expression level, respectively. RT-qPCR experiment was repeated three times with each sample having three technique replicates. The relative expression level of genes was calculated using the 2^−ΔΔC*T*^ method, and standard deviation was calculated between three biological replicates [13].

### 2.9. Statistical Analysis

The data were analyzed by the analysis of variance (ANOVA) using the statistical program SPSS/PC version17.0 (SPSS Inc., Chicago, IL, USA) and the Duncan’s multiple range test was used for separation of means. The statistical significance was applied at the level of *p* < 0.05.

## 3. Results

### 3.1. Infection of Apples by P. expansum

**Figure 1:**
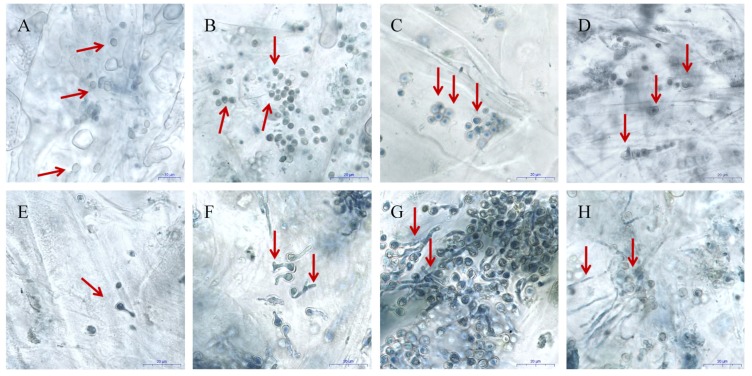
Microscopic images of the activation, germination, and growth of *P. expansum* spores and germ tubes at 25 °C in wounded apple fruit tissue. (**A**–**C**) *P. expansum* spores in wounded apple tissue at 0, 1, and 3 h post-inoculation (hpi). (**D**,**E**) *P. expansum* spores and germ tubes at 6 hpi. (**F**,**G**) *P. expansum* spores and germ tubes at 9 hpi. (**H)** Spores, germ tubes and young mycelia of *P. expansum* in wounded apple tissues at 12 hpi. Scale bar represents in A–H represents 20 µm. Red rows represent the typical form of *P. expansum* at different time points.

The spores of *P. expansum* were oval or round when initially inoculated into wounded apple tissues (Figure 1A). The spores became activated at 1 hpi exhibiting irregular shapes that were distinctly different from their original shape (Figure 1B). The spores swelled at 3 hpi (Figure1C), and then germinated with germ tube penetration of the apple tissue visible at 6 hpi (Figure 1D,E). The developing germ tubes penetrated into the apple tissues (Figure 1F) and continued to proliferate in the wounded apple tissue (Figure 1G). The primary mycelia continued to extend into the apple tissues and were easily observed by12 hpi (Figure 1H). Based on these observations, samples of apple tissue, along with the resident spores, germ tubes, and mycelia of *P. expansum* were collected at 1, 3, and 6 hpi and subjected to RNA-seq analysis using 0 hpi as a control.

### 3.2. RNA-Seq Analysis and Validation

An RNA-seq analysis was conducted to determine the changes in gene expression that occurred in both apple tissues and *P. expansum* over the first 6 h of the infection process. In Table S2, the Illumina sequencing of the prepared libraries provided 46.38 Gb of clean sequence data where the Q30 was 89.71%, 88.84%, 90.60% and 91.82% for the reads representing the four time points (0, 1, 3, 6 hpi), respectively (Table S2). These data indicate that the sequencing provided a sufficient number of high-quality reads that were used in the subsequent analyses. The obtained sequence data were submitted to the NCBI’s Gene Expression Omnibus (GEO) public archive database under the accession number GSE119039.

**Figure 2:**
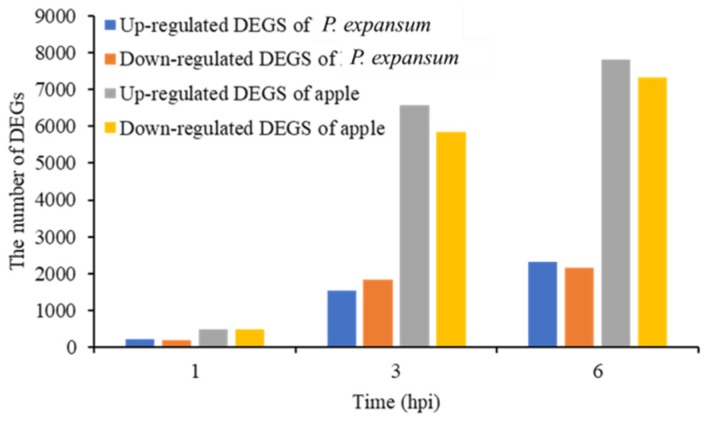
The total number of up- and down-regulated *P. expansum* and apple differentially expressed genes (DEGs) at 1 h post-inoculation (hpi), 3 hpi, and 6 hpi.

Figure 2 illustrates the number of DEGs in *P. expansum* and apple tissues that were identified at 1, 3, and 6 hpi. A **|**Log2 fold-change**|** ≥1 in the level of gene expression and an FDR < 0.05 was used to define a significant difference in the level of gene expression. A total of 200 DEGs were up-regulated and 186 DEGs were down-regulated in *P. expansum* at 1 hpi, relative to 0 hpi. A total of 1528 DEGs were up-regulated and 1839 DEGs were down-regulated in *P. expansum* at 3 hpi. A total of 2306 DEGs were up-regulated and 2158 DEGs were down-regulated in *P. expansum* at 6 hpi. All comparisons in gene expression are relative to 0 hpi. A total of 386, 3387, and 4464 DEGs were identified in *P. expansum* at 1, 3, and 6 hpi, respectively.

DEGs were also identified in apple tissues at 1 hpi relative to the control (0 hpi), 473 DEGs were up-regulated and 471 DEGs were down-regulated. Subsequently, 6576 DEGs were up-regulated and 5853 DEGs were down-regulated at 3 hpi, 7805 DEGs were up-regulated and 7317 DEGs were down-regulated at 6 hpi. All comparisons in gene expression were again relative to the level of expression at 0 hpi. A total of 944, 12,429, and 15,122 DEGs were identified in apple tissues at 1, 3, and 6 hpi, respectively.

The results indicate a progressively greater number of DEGs in *P. expansum* and in wounded fruit tissues relative to the level of expression at 0 hpi (control), as post-inoculation time progressed. More DEGs were identified in apple than in *P. expansum* during the early stages of infection with the number of DEGs increasing sharply in both *P. expansum* and apple at 3 hpi. These results suggest that major host and pathogen responses are occurring between 0–3 hpi. Although the number of DEGs was lower at 1 hpi relative to 3 and 6 hpi, the DEGs identified at this time may play a critical role in the early infection process, prior to the proliferation of *P. expansum* and extensive invasion of hyphae into apple tissue.

**Figure 3:**
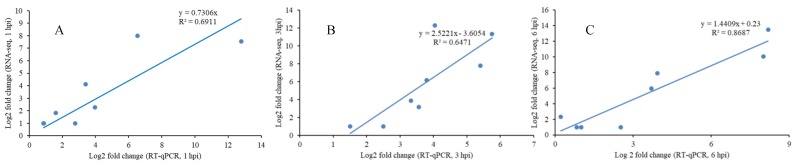
The comparison of gene expression values obtained by RNA-seq and RT-qPCR at 1 hpi, 3 hpi and 6 hpi. (**A**) 1 hpi; (**B**) 3 hpi; (**C**) 6 hpi. The RNA-seq log2 values of the expression ratio (infected versus uninfected) (*x*-axis) was compared to the same values from the RT-qPCR (*y*-axis). The R2 values show the correlation ratio between RNA-seq and RT-qPCR in each time point. *p*-value ≤ 0.05.

To confirm the accuracy of the results obtained in the transcriptomic analysis, ten DEGs were randomly selected for RT-qPCR analysis. The relative fold-change in the expression level of genes obtained by RT-qPCR was converted to log2 (fold-change) to compare the results with the RNA-seq data. Results indicated that the pattern of up- and down-regulation of the selected genes were similar in both the RT-qPCR and RNA-seq data (Figure 3). The gene-specific primers used in the RT-qPCR analysis are listed in Table S1.

### 3.3. Gene Expression Pattern Analysis, Clustering and Functional Enrichment of DEGs

**Figure 4:**
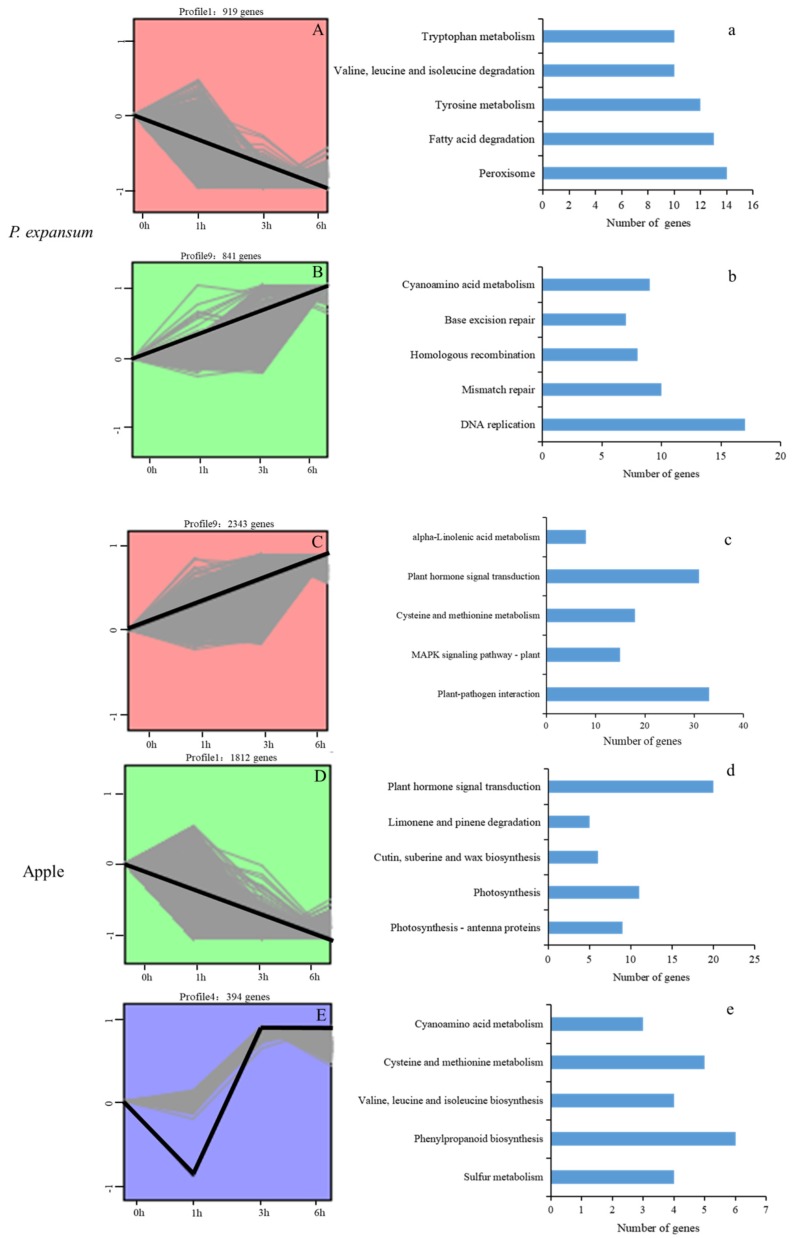
Patterns of gene expressions (**A**–**E**) and the top 5 KEGG enrichment pathway categories (**a**–**e**) of the DEG profiles of *P. expansum* and apple which were significantly different from the reference set (DEGs at 0 hpi) combined across three time points (1, 3, and 6 hpi). The light grey lines in each figure represent the expression pattern of an individual gene, while the black line represents the average expression pattern of all the genes combined. The number of genes represented in each profile is indicated above each figure.

The DEGs in *P. expansum* and apple that exhibited considerable differences in expression during the early stages of the infection process is shown in Figure 4. The number of DEGs in the significantly different expression profiles were very different in *P. expansum* and apple. The DEGs in *P. expansum* were significantly overrepresented in the profiles with opposite levels of expression in Profile 1 and 9. Profile 1 (919) exhibits down-regulated patterns of expression and profile 9 (841) exhibits an up-regulated pattern of expression. Notably, the major transcriptional changes in apple were present in Profile 9 (2343), 1 (1812), and 4 (394). These results suggest that there is a greater response at the transcriptional level in apples, rather than in *P. expansum* during the early stages of infection.

KEGG pathway enrichment analysis of the DEGs in the overrepresented profiles was conducted to investigate the function of the transcriptional changes in *P. expansum* and apple. DEGs involved in peroxisome, fatty acid degradation, tyrosine metabolism, valine, leucine and isoleucine degradation, as well as tryptophan metabolism were the top five enriched categories in profile 1 in *P. expansum*, (Figure 4a), and exhibited a decreasing trend in expression from 0 to 6 hpi (Figure 4A). In contrast, the overrepresented pathways in profile 9 exhibited an increasing trend in expression from 0 to 6 hpi (Figure 4B), and included DNA replication, mismatch repair, homologous recombination, base excision repair, and cyanoamino acid metabolism (Figure 4b). In apple, however, DEGs involved in plant-pathogen interaction, cysteine and methionine metabolism, MAPK signaling pathway—plant, plant hormone signal transduction, and alpha-Linolenic acid metabolism were the top five overrepresented categories in Profile 9 (Figure 4c), where the level of gene expression exhibited an increasing trend from 0 to 6 hpi (Figure 4C). While DEGs related to the categories of photosynthesis—antenna proteins, photosynthesis, cutin, suberin and wax biosynthesis, limonene and pinene degradation, and plant hormone signal transduction were enriched in Profile 1 (Figure 4d), where the level of gene expression exhibited a decreasing trend from 0 to 6 hpi (Figure 4D). The overrepresented DEGs in profile 4 decreased from 0 to 1 hpi, then increased and peaked at 3h, and finally maintained a relatively high level of expression (Figure 4E). DEGs related to cyanoamino acid metabolism, cysteine and methionine metabolism, valine, leucine and isoleucine degradation, phenylpropanoid biosynthesis, and sulfur metabolism were enriched in Profile 4 (Figure 4e). These results indicate a distinct variation in the pattern of DEG expression and KEGG pathway categories in *P. expansum* vs. apple during their interaction. The differences in the pattern and functional categories of gene expression between *P. expansum* and apple reflect their distinct responses to each other during the infection process.

### 3.4. Major DEGs in P. expansum Identified During the Early Infection Process in Apple

The key DEGs expressed in *P. expansum* during the early stages of the infection process in apples, based on GO and KEGG enrichment analysis are listed in Tables S3–S5. They comprise of genes belonging to cell wall degrading enzymes (CWDEs), anti-oxidative stress, pH regulation, and effectors.

CWDEs are important virulence factors when pathogens invade host tissues [14]. The DEGs related to CWDEs are listed in Table S3. These CWDEs fell into four major categories: Cellulase, hemicellulase, pectinase, and uncharacterized hydrolase. A total of 3, 13, and 32 up-regulated CWDEs were identified at 1, 3 and 6 hpi, respectively. Cellulase (PEX2_048700), hemicellulase (PEX2_011360), and uncharacterized hydrolase activity (PEX2_082040) genes were up-regulated at 1 hpi, pectinases (PEX2_094840, PEX2_016450, PEX2_040830, PEX2_013190) were activated at 3 hpi. Most of the CWDEs were highly up-regulated at 6 hpi if they had been induced at 1 and 3 hpi. In addition to the identification of other CWDEs that were induced at 6 hpi, most of the CWDEs induced at 1 and 3 hpi were also highly up-regulated at 6 hpi. PEX2_082040 was the only up-regulated DEG related to CWDEs that was detected at all examined time points. The fold change of PEX2_082040 increased sharply from 1 hpi (2.27-fold) to 3 hpi (7.80-fold) and continued to increase till 6 hpi (7.92-fold). This gene encodes a precursor of alkali-sensitive linkage protein 1 and contains a glycosyl hydrolase catalytic core conserved domain which may alter the redox status in *P. expansum* and affect its ability to infect host tissues and result in a rapid adaptation to a stressful redox environment to determining the establishment of infection in the apple-*P. expansum* pathosystem [8]. Thus, PEX2_082040 may play a vital catalytic role in the apple cell wall degrading during the early stages of the infection of apple by *P. expansum*.

The accumulation of reactive oxygen species (ROS) in host tissues can inhibit or prevent the establishment of a pathogen [15]. Thus, pathogens have developed several defense mechanisms to resist ROS, including the production of antioxidant enzymes and increased resistance to non-enzymatic protective molecules, such as thioredoxin [16]. The RNA-seq analysis of *P. expansum* identified four glutathione S-transferases (PEX2_063170, PEX2_007300, PEX2_109060, PEX2_011570) and 1 catalase (PEX2_018990), all of which function in preventing oxidative stress (Table S4). The five genes were not expressed at 1 hpi, but were expressed at 3 hpi or 6 hpi. PEX2_063170, PEX2_007300 and PEX2_018990 were significantly up-regulated at 3 hpi, while PEX2_109060 and PEX2_011570 were significantly up-regulated at 6 hpi.

The regulation of pH is also a method that can be used to affect the establishment of a pathogen in host tissues [17]. Glucose oxidase can catalyze glucose to produce gluconic acid that can change the pH in host tissues and offer a more beneficial pH environment for the activity of CWDEs [18]. PEX2_108150 is a glucose-methanol-choline oxidoreductase and the precursor of glucose oxidase. While it was not expressed at 1 hpi, it was highly up-regulated (7.46-fold) at 3 hpi and increased to 8.20-fold at 6 hpi, relative to 0 hpi. This indicates that PEX2_108150 plays an important role in the apple cell wall degrading during the early infection of apple tissues by *P. expansum*.

Host tissues can activate a systemic defense system in response to the attack of a pathogen, which then responds by secreting different effectors into the cytoplasm of host cells or extracellular space of host tissues [19]. Effectors not only directly induce host cell death, but also inhibit or prevent the recognition of the pathogen by the host. Thus, the host does not activate a defense response, which allows the pathogen to colonize the host [20]. In the current study, seven DEGs were classified as three different kinds of effectors (necrosis inducing protein, chitinase, and LysM) (Table S5). PEX2_044440, PEX2_110230, and PEX2_034750 were activated at 3 hpi, and their expression increased at 6 hpi, while the differential expression of PEX2_080220, MSTRG.2227, and PEX2_020570 were detected at 6 hpi.

### 3.5. Major DEGs in Apple

**Figure 5:**
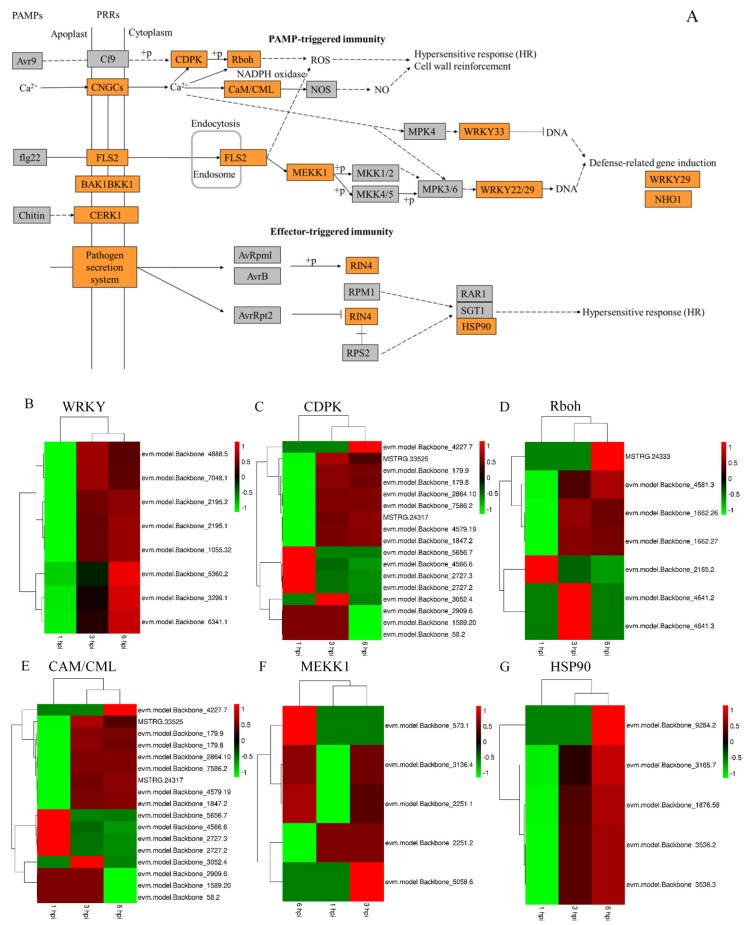
Major DEGs and their pattern of expression identified at 1, 3 and 6 hpi in wounded apple fruit tissues inoculated with *P. expansum* in the context of metabolic pathway analysis. (**A**) DEGs identified in the context of an apple-*P. expansum* interaction metabolism pathway. The metabolites, transporter proteins, and transcriptional factors that were DEGs are indicated in orange boxes, while the other metabolites in the metabolic pathway are indicated in gray boxes. Arrows made from hashed lines indicate multiple potential enzyme reactions that are not relevant to the present study. Arrows pointing towards the orange boxes represent the direction of signal transport for the transporter genes. Arrows pointing away from a transcription factor represent the resulting products induced by the transcription factor. (**B**–**G**) Heat maps of apple DEGs associated with PAMP-triggered immunity (PTI) and effector-triggered immunity (ETI). (**B**) WRKY. (**C**) CDPK. (**D**) Rboh. Calmodulin (CAM)/ calmodulin-like (CML). (**F**) MEKK1. (**G**) HSP90. Colors ranging from bright green (down-regulation) to bright red (up-regulation) represent the level of expression.

*P. expansum* infection of apple induces the expression of genes that play a role in defense response, hypersensitive response, and cell wall reinforcement, all of which play an important role in defense against pathogens (Figure 5A). The results of the RNA-seq analysis indicated that the infection by *P. expansum* spores induced the expression of *WRKY29* (defense-related genes) at 1 hpi. The expression of WRKY22 and WRKY33 transcription factors were up-regulated as spores began to swell at 3 hpi, and the expression level of most of the identified WRKY transcriptional factors increased as fungal mycelia began to invade apple host tissues at 6 hpi. These data support the idea that *P. expansum* infection triggers weak defense responses in apple and that the WRKY29 transcription factors represents an early defense-related gene in apple (Figure 5B).

Calcium influx is required either for ‘nutritional’ or ‘signaling’ purposes in plant cells [21]. Calcium signaling pathways play an important role in sensing internal and external stimuli and transducing them into changes in gene expression and physiology [21]. Cyclic nucleotide-gated channels (CNGCs) have been postulated to play a significant functional role in plant development and stress resistance [22]. As illustrated in Figure 5C, calcium is transported through the CNGCs into apple cells and activates CDPK (calcium-dependent protein kinase)-Rboh (Respiratory burst oxidase homolog) to generate ROS accumulation (Figure 5C,D). A hypersensitive response (HR) is also activated in response to *P. expansum.* The calcium influx into apple cells also induces calmodulin (CAM) and calmodulin-like (CML) proteins that regulate NO synthase (NOS) activity, the latter of which plays a role in cell wall reinforcement (Figure 5E). The calcium influx indirectly mediates the activation of WRKY transcription factors.

As shown in Figure 5A, the plant immune receptor gene, FLS2 (Flagellin Sensing 2), can activate the plant MAP kinase cascade, MEKK1 (Figure 5F), which can then activate WRKY22/WRKY29 transcription factors. Activation of the MAPK cascade induces defense-related gene expression. Chitin elicitor receptor kinase (CERK1) is essential for the activation of chitin-triggered innate immunity [23]. Figure 5A illustrates that CERK1 is also activated by *P. expansum.* Effectors secreted by the pathogen will trigger plant immunity (ETI). In this study, the expression of the RPM1-interacting protein 4 (RIN4) gene and a 90-kDa heat shock protein (HSP90) gene were induced by the presence of *P. expansum* (Figure 5G).

## 4. Discussion

Blue mold, caused by *P. expansum,* is a major postharvest disease of apples [24]. Fungal infection is usually initiated by contact of spores with host tissues which then triggers spore germination [25]. Activation of spores is evident with the isotropic growth of spores, which is a morphological change termed, swelling [25]. Swelling is accompanied by cell wall expansion, water uptake, changes in cellular composition, and a decrease in cytoplasmic micro-viscosity [26]. After spores swell, cell wall deposition of polysaccharides, such as chitin, occurs in a polar fashion, and the extension of the fungal cell occurs at a restricted area at the tip of the cell [25]. Though the genomes of *P. expansum* were sequenced at 2015 [8]. However, there is still currently limited information on transcriptome studies on the interaction between *P. expansum* and apple during the early stages of infection (before the germ tube elongation stage). Results of the current study revealed that the activation stage (1 hpi), swelling stage (3 hpi), and germ tube elongation stage (6 hpi) represent pivotal time points in the early infection process of *P. expansum*. Therefore, RNA-seq analysis was then conducted to determine changes in gene expression in both *P. expansum* and apple at these critical time points (1, 3 and 6 hpi) in the infection process. Ballester et al. investigated the transcriptomics of the *P. expansum* at 24, 48, and 72 h. The DEGs in *P. expansum* were increased with time which keeps the same trend with the result of our study [8]. The RNA-seq analysis conducted at 1, 3, and 6 hpi demonstrated that the transcriptional data obtained was accurate and reliable.

The gene expression in *P. expansum* and apple fruit tissues during the early stages of infection contributes to a better understanding of the host-pathogen interaction. Peroxisomes perform many essential functions in eukaryotic cells and mediate fungal growth and survival [27]. Fatty acid degradation, tyrosine metabolism, valine, leucine and isoleucine degradation, and tryptophan metabolism are essential to basic metabolism. Cyanoamino acid metabolism functions in a potential detoxification pathway in *Bacillus GJ1* [28]. The activation of detoxification processes was also found at 5 days post *P. expansum* spores inoculation on apple at 25 °C [29]. Therefore, the gene expression pattern of *P. expansum* DEGs in profile 1 and 9 indicated that *P. expansum* gradually focused on the detoxification and reproduction process. The KEGG and GO enrichment analysis suggests that *P. expansum* absorbed apple nutrients (basic metabolism), adapted to the environment (response to stimulus), and prepared for the penetration of apple wound tissues during the initial stages (0 to 6 hpi) of the infection process.

Plants respond to pathogens through the activation of an immune system, referred to as pathogen-associated molecular pattern (PAMP)-triggered immunity (PTI), effector-triggered immunity (ETI), and phytohormone signaling [30]. Both PTI and plant hormone signal transduction were activated by the presence of *P. expansum* in wounded apple fruit tissues at the spore activation stage (1 hpi). ETI in apple fruit tissues, however, was activated at the spore swelling stage (3 hpi). All three immunity systems were more highly induced at the germ tube intrusion stage (6 hpi) than at the earlier stages (0 to 3 hpi) of the infection process.

SA-inducible defense responses are regulated by MAPK and EDR1 [31]. MAPKs can also directly activate defense genes by the phosphorylation of downstream transcription factors. *WRKY29* was activated in apple fruit tissues at 1 hpi. The expression of apple DEGs in profile 9 indicated that plant-pathogen interaction, MAPK signaling pathway–plant, and plant hormone signal transduction were activated in apples, in response to *P. expansum* infection.

CWDEs are one of the most important pathogenic factors in postharvest pathogens, and are responsible for degrading the cell wall or secondary metabolites of the host, thus, causing the softening and decay of fruits [32]. These enzymes include pectinases, cellulases, hemicelluloses, and hydralases. Most of the identified CWDEs in this research were members of the glycoside hydrolase family. Glycoside hydrolases (GH) function in the biosynthesis of glycans, signaling, plant defense, and the mobilization of storage reserves in cell wall metabolism [33]. Our data suggest that CWDEs play an important role in the early stages of the infection process.

Glutathione peroxidase not only mediates ROS resistance, fungicide sensitivity, and cell wall construction in *Alternaria lternate*, but also regulates the efficient use of oxidative stress. A glutathione peroxidase 3 (AaGPx3) was identified as a component of a signaling network and play an important role in responding to cellular stresses induced by ROS in *A. lternate* in citrus [34]. The different timing (3 vs. 6 hpi) in the expression of these antioxidant genes in this research may be related to differences in their functional role in dealing with oxidative stress.

Changes in the pH of the micro-environment of a pathogen has a strong effect on the infection process [35]. Pathogens increase their virulence by regulating the pH of the microenvironment within their host [35]. Glucose oxidase can catalyze glucose to produce gluconic acid, and gluconic acid can change the pH in host tissues. *P. expansum* has been reported to be an “acid fungus”, and secretes CWDEs in apple and other hosts through acidification of their micro-environment [29]. The high expression of PEX2_108150 is similar to the trend reported in previous studies [36].

Multicellular organisms activate immunity systems upon recognition of PAMPs [37]. Chitin is an important component of fungal cell walls, and chitin oligosaccharides act as PAMPs in plant and mammalian cells [38]. Pathogens produce effector proteins to suppress PTI, which allows them to establish an infection. Data in the current study identified necrosis inducing protein, chitinase, and some effectors containing a LysM domain asup-regulated DEGs. Necrosis inducing protein not only directly induces cell death in host tissues, but also inhibits host defense response, which benefits the colonization of host tissue by the pathogen [39]. Effectors containing a LysM domain mediate virulence by perturbation of chitin-triggered host immunity. During the infection process, the cell walls of invading hyphae release Ecp6 sequesters, which binds chitin oligosaccharides, and prevent them from triggering host immunity. This may represent a common strategy of host immune suppression by fungal pathogens, because LysM effectors are widely conserved in the fungal kingdom [39]. The up-regulated expression of effectors in *P. expansum* may trigger ETI in apple fruit tissues.

**Figure 6:**
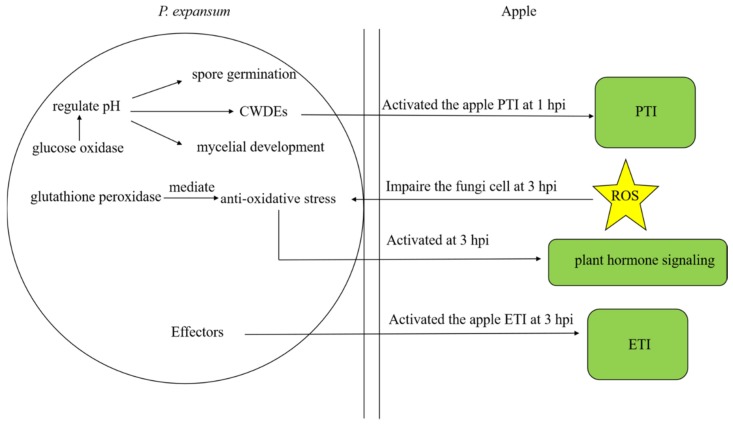
The interaction analysis between *P. expansum* and wounded, inoculated apple fruit tissues. Glucose oxidase expression by *P. expansum* altered the pH of the micro-environment, providing an optimum pH for spore germination, mycelial development, and cell wall degrading enzyme (CWDE) activity. CWDEs expressed by *P. expansum* activated (PAMP)-triggered immunity (PTI) in apple at 1 hpi. Apple tissues generated an accumulation of ROS to impair pathogen cells at the swelled spore stage (3 hpi) of *P. expansum*. The expression of glutathione peroxidase was induced in *P. expansum* to mediate oxidative stress. PTI was activated in apple at the spore activation stage (1 hpi), then effector-triggered immunity (ETI) and plant hormone signaling were activated at the swelled spore stage (3 hpi) in response to the presence of *P. expansum*.

Results indicated that the time courses of 1, 3, and 6 hpi are important time points during the early stages of the infection of wounded apple fruit tissues by *P. expansum.* RNA-seq analysis of the transcriptome of *P. expansum* and wounded, inoculated apple fruit tissues revealed a complex set of changes in gene expression that occur during the early stages of the infection process (Figure 6). CWDEs expressed by *P. expansum* activated PAMP-triggered immunity (PTI, Defense-related gene induction) at 1 hpi. Apple tissues generated an accumulation of ROS to impair pathogen cells at the swelled spore stage (3 hpi) of *P. expansum.* In response to the elevated levels of ROS, expression of glutathione peroxidase was induced in *P. expansum* to mediate oxidative stress. Glucose oxidase expression by *P. expansum* altered the pH of the micro-environment, providing an optimum pH for spore germination, mycelial development, and CWDE activity. PTI was activated in apple at the spore activation stage (1 hpi), then effector-triggered immunity (ETI) and plant hormone signaling were activated at the swelled spore stage (3 hpi) in response to the presence of *P. expansum*.

## Supplementary Materials

The following are available online at https://www.mdpi.com/2076-2607/7/11/495/s1.
