# Supplementary material for: Comparative Transcriptomic Analysis of the Interaction between Penicillium expansum and Apple Fruit (Malus pumila Mill.) during Early Stages of Infection
Source: Microorganisms. 2019 Oct 28;7(11):495. doi: 10.3390/microorganisms7110495 (PMC6920851; doi:10.3390/microorganisms7110495)
Supplement: Supplementary file 1 [file microorganisms-07-00495-s001.pdf]

# Comparative Transcriptomic Analysis of the Interaction between *Penicillium expansum* and Apple Fruit（*Malus pumila Mill.*）during Early Stages of Infection

**Kaili Wang , Xiangfeng Zheng, Xiaoyun Zhang, Lina Zhao, Qiya Yang, Nana Adwoa Serwah Boateng, Joseph Ahima, Jia Liu and Hongyin Zhang**

**Table S1.** Gene specificity primers used in this study.

| Gene No. | Primer | Primer Sequence (5' to 3') |
| --- | --- | --- |
| PEX2_082040 | GHS-F | ATTCTGGGATTCAACGAGC |
| | GHS-R | GGTGGAGGAGGTTACGG |
| PEX2_082310 | TFS-F | AATTTGTTCCGCCTCTTCG |
| | TFS-R | GTTGACCCGTCTTGTAGCC |
| PEX2_073880 | GH32-F | CCGAAGTAAACCACGGCTATGAA |
| | GH32-R | CGTTTCTTCAACCTCTTCGCCAC |
| PEX2_064810 | GRG1-F | GTCGCCAAGGACTCTGA |
| | GRG1-R | GCCACAACCGTTCTATTT |
| β-tublin | β-tublin-F | AGCGGTGACAAGTACGTTCC |
| | β-tublin-R | ACCCTTGGCCCAGTTGTTAC |
| evm.model.Backbone_6341.1 | 6341.1-F | GCCACATCCGAGGAGCTATTACAG |
| | 6341.1-R | CGACGAGTTGGATGAACATGGTTG |
| evm.model.Backbone_2586.16 | 2586.16-F | CGCAGACCAACGCCAGGAAG |
| | 2586.16-R | ACTGAGGCATGATGGACATTACGC |
| evm.model.Backbone_2503.31 | 2503.31-F | AATTGGTGCTCAGGTGGTGATTCC |
| | 2503.31-R | GGTCCGCCTGCGGTTGAATG |
| evm.model.Backbone_1662.27 | 1662.27-F | CTCAAGTTCATCACCGCCAAGACC |
| | 1662.27-R | GGAGGAGTGGAGGAGCCAGTTG |
| MdActin | MdActin-F | CCCAAAGGCTAATCGGGAGAAA |
| | MdActin-R | ACCACTGGCGTAGAGGGAAAGA |

**Table S2.** Statistic results of sequencing.

| Time (hpi) | Raw Reads | Clean Reads | Clean Base(G) | Error Rate(%) | %≥Q20 | %≥Q30 |
| --- | --- | --- | --- | --- | --- | --- |
| 0 | 45426166 | 44788180 | 13.44 | 0.03 | 95.63 | 89.71 |
| 1 | 42462649 | 41897109 | 12.57 | 0.04 | 95.22 | 88.84 |
| 3 | 36419573 | 35664992 | 10.70 | 0.02 | 96.42 | 90.60 |
| 6 | 33107420 | 32230326 | 9.67 | 0.02 | 96.83 | 91.82 |

**Table S3.** The up-regulated DEGs related to CWDEs at three time points.

| CWDEs | Function Classfication | Gene id | Gene Description | Fold Change | | |
|---|---|---|---|---|---|---|
| | | | | **1 hpi** | 3 hpi | 6 hpi |
| cellulase | xyloglucan-specific endo-beta-1,4-glucanase activity | PEX2_072090 | Glycoside hydrolase, family 12 | 1 | 1.85 | 1 |
| | xyloglucan-specific endo-beta-1,4-glucanase activity | PEX2_059230 | Glycoside hydrolase, family 12 | 1 | 6.59 | 7.29 |
| | Glucan 1,4-alpha-glucosidase activity | PEX2_048700 | Six-hairpin glycosidase | 1.92 | 2.18 | 1 |
| | cellulase activity | PEX2_013740 | Protein of unknown function DUF2985 | 1 | 4.50 | 3.91 |
| | cellulase activity | PEX2_020130 | Glycoside hydrolase, family 12 | 1 | 2.46 | 3.20 |
| | Beta-glucosidase | PEX2_032420 | Uncharacterized protein | 1 | 3.59 | 7.37 |
| | cellulase activity | PEX2_056710 | Glycoside hydrolase, superfamily | 1 | 1 | 1.96 |
| | glucan exo-1,3-beta-glucosidase activity | PEX2_093000 | Glycoside hydrolase, superfamily | 1 | 1 | 2.11 |
| | alpha-1,4-glucosidase activity | PEX2_015850 | Glucosidase 2 subunit beta | 1 | 1 | 1.68 |
| | glucan endo-1,3-beta-D-glucosidase activity | PEX2_071750 | Glycoside hydrolase, family 81 | 1 | 1 | 2.67 |
| | alpha-1,4-glucosidase activity | PEX2_030530 | Glycoside hydrolase, superfamily | 1 | 1 | 2.26 |
| | alpha-1,4-glucosidase activity | PEX2_097510 | Glycoside hydrolase, family 31 | 1 | 1 | 2.07 |
| | alpha-1,4-glucosidase activity | PEX2_000950 | Glycoside hydrolase, family 31 | 1 | 1 | 1.84 |
| | beta-glucosidase activity | PEX2_004230 | Glycoside hydrolase family 3 | 1 | 1 | 6.45 |
| | beta-glucosidase activity | PEX2_092420 | Glycoside hydrolase, family 1 | 1 | 1 | 6.30 |
| hemicellulase | xylan 1,4-beta-xylosidase activity | PEX2_011360 | Glycoside hydrolase, superfamily | 2.80 | 1 | 1 |
| | mannan endo-1,4-beta-mannosidase activity | PEX2_081690 | Glycoside hydrolase, superfamily | 1 | 3.37 | 1 |
| | mannan endo-1,4-beta-mannosidase activity | PEX2_088710 | Glycoside hydrolase, superfamily | 1 | 2.79 | 2.65 |
| | mannosyl-oligosaccharide 1,2-alpha-mannosidase activity | PEX2_104840 | Glycoside hydrolase, family 47 | 1 | 1 | 1.55 |
| | arabinan endo-1,5-alpha-L-arabinosidase activity | PEX2_089180 | Glycoside hydrolase, family 43 | 1 | 1 | 2.92 |
| | arabinan endo-1,5-alpha-L-arabinosidase activity | PEX2_004580 | Glycoside hydrolase, family 43 | 1 | 1 | 6.71 |
| | alpha-L-arabinofuranosidase activity | PEX2_068560 | Glycoside hydrolase, superfamily | 1 | 1 | 2.30 |
| | mannosyl-oligosaccharide | PEX2_025180 | FAD dependent | 1 | 1 | 1.27 |

| | | | | | | |
|---|---|---|---|---|---|---|
| | glucosidase activity | | oxidoreductase | | | |
| pectinase | polygalacturonase activity | PEX2_094840 | Glycoside hydrolase, family 28 | 1 | 3.65 | 7.13 |
| | polygalacturonase activity | PEX2_013180 | Glycoside hydrolase, family 28 | 1 | 1 | 1.96 |
| | pectate lyase activity | PEX2_016450 | Pectin lyase fold/virulence factor | 1 | 1.76 | 1.41 |
| | pectate lyase activity | PEX2_007290 | Pectate lyase, catalytic | 1 | 1 | 2.63 |
| | pectinesterase activity | PEX2_040830 | Pectinesterase, catalytic | 1 | 5.68 | 5.35 |
| | pectinesterase activity | PEX2_013190 | Pectinesterase, catalytic | 1 | 6.59 | 6.58 |
| uncharacterized hydrolase | Alkali-sensitive linkage protein 1 (Precursor) | PEX2_082040 | Glycoside hydrolase, superfamily | 2.27 | 7.80 | 7.92 |
| | Probable beta-glucosidase G (Precursor) | PEX2_030850 | Glycoside hydrolase family 3 | 1 | 1 | 8.11 |
| | Probable beta-glucosidase I | PEX2_015940 | Glycoside hydrolase family 3 | 1 | 1 | 5.74 |
| | Probable beta-glucosidase btgE (Precursor) | PEX2_066430 | Glycoside hydrolase, superfamily | 1 | 1 | 3.78 |
| | Probable beta-glucosidase I | PEX2_032330 | Glycoside hydrolase family 3 | 1 | 1 | 8.75 |
| | Glucan endo-1,3-alpha-glucosidase agn1 (Precursor) | PEX2_056100 | Glycoside hydrolase, family 71 | 1 | 1 | 2.20 |
| | Glucan endo-1,3-alpha-glucosidase agn1 (Precursor) | PEX2_054130 | Glycoside hydrolase, family 61 | 1 | 1 | 6.45 |

**Table S4.** The up-regulated DEGs related to anti-oxidative stress at three time points.

| **Anti-oxidative stress** | **anti-oxidative stress** | **Gene id** | **Gene description** | **Fold change** | | |
|---|---|---|---|---|---|---|
| | | | | **1 hpi** | 3 hpi | 6 hpi |
| Glutathione S-transferase | transferase activity | PEX2_063170 | Glutathione S-transferase, N-terminal | 1 | 3.15 | 3.05 |
| | transferase activity | PEX2_007300 | Glutathione S-transferase, N-terminal | 1 | 2.80 | 3.11 |
| | transferase activity | PEX2_109060 | Glutathione S-transferase, N-terminal | 1 | 1 | 2.13 |
| | transferase activity | PEX2_011570 | Glutathione S-transferase/chloride channel, C-terminal | 1 | 1 | 1.66 |
| Catalase | catalase activity | PEX2_018990 | Catalase, mono-functional, heme-containing | 1 | 4.85 | 4.52 |

**Table S5.** The up-regulated effectors related to infection process at three time points.

| Effectors | Function classfication | Gene id | Gene description | Fold change 1 hpi | 3 hpi | 6 hpi |
|---|---|---|---|---|---|---|
| Necrosis inducing protein | Necrosis inducing protein | PEX2_080220 | Necrosis inducing protein | 1 | 1 | 6.15 |
| chitinase | chitinase activity | PEX2_044440 | Endochitinase A (Precursor) | 1 | 5.11 | 7.53 |
| | chitinase activity | PEX2_110230 | Alpha/beta hydrolase family | 1 | 2.08 | 2.39 |
| | chitinase activity | PEX2_025780 | Peptidoglycan-binding Lysin subgroup | 1 | 1 | 4.49 |
| LysM | oxidoreductase activity | PEX2_034750 | LysM domain | 1 | 1.97 | 2.27 |
| | LysM domain | MSTRG.2227 | LysM domain | 1 | 1 | 3.94 |
| | Signal transduction | PEX2_020570 | Peptidoglycan-binding Lysin subgroup,LysM domain | 1 | 1 | 1.85 |
